# Orthodontic Treatment for a Patient with Root Resorption of All Four Maxillary Incisors due to Bilaterally Impacted Canines

**DOI:** 10.1155/2022/5628030

**Published:** 2022-09-19

**Authors:** Makoto Yanoshita, Naoto Hirose, Azusa Onishi, Sayuri Nishiyama, Naoki Kubo, Daiki Kita, Kotaro Tanimoto

**Affiliations:** Department of Orthodontics and Craniofacial Developmental Biology, Hiroshima University Graduate School of Biomedical and Health Sciences, Kasumi 1-2-3 Minami-ku, Hiroshima-shi, Hiroshima Prefecture, Japan

## Abstract

Maxillary canines require the longest period from the generation of the tooth germ to the completion of eruption, and they have more difficulties in eruption than other teeth. The incisor roots are often resorbed due to malpositioned canines. An 11-year-old girl presented with an extremely rare case of root resorption of four maxillary incisors due to bilaterally impacted maxillary canines, which were located excessively mesially. The case was managed through oral surgery and orthodontic treatment over five years. After extracting the maxillary deciduous canines, the maxillary bilateral canines were surgically exposed. The bimaxillary lateral incisors were extracted, and the canines were orthodontically tracted over 8 months. All teeth were then aligned using edgewise brackets. No further root resorption of the central incisors was observed for 5 years after canine traction. This case demonstrates the importance of early detection of abnormally positioned canines.

## 1. Introduction

Maxillary canines are important in terms of function and esthetics. Canines have the greatest frequency of impaction, second only to third molars [1, 2]. There is a 1–3% chance that canines fail to erupt and become impacted [3]. The period between the formation of the tooth germ and completion of tooth eruption is the longest for maxillary canines; they also experience more difficulty in eruption compared with other teeth [3]. The causes of impaction of maxillary canines include crowding, trauma, root curvature, odontoma, cysts, and supernumerary teeth [4–8].

Impacted canines can cause root resorption in permanent incisors. This root resorption is assumed to be caused by physical contact between the incisor roots and canine crown [9]. Root resorption associated with ectopic eruption of maxillary canines occurs in 48 of patients with impacted canines aged 9 to 15 years [10].

Deciduous root resorption is a normal physiological process that precedes permanent tooth eruption. Root resorption of permanent teeth is considered the result of complex biological processes; however, the exact mechanism remains unclear. In case of root resorption of the permanent maxillary incisors due to an impacted maxillary canine, prompt decisions must be made regarding the need for surgical exposure of the canine, traction of the canine, and extraction of the resorbed incisors should be extracted, followed by orthodontic alignment.

This report describes the surgical and orthodontic management of a case of root resorption of the bilateral maxillary central and lateral incisors due to maxillary canines erupting in an abnormal direction.

## 2. Case Report

**Figure 1:**
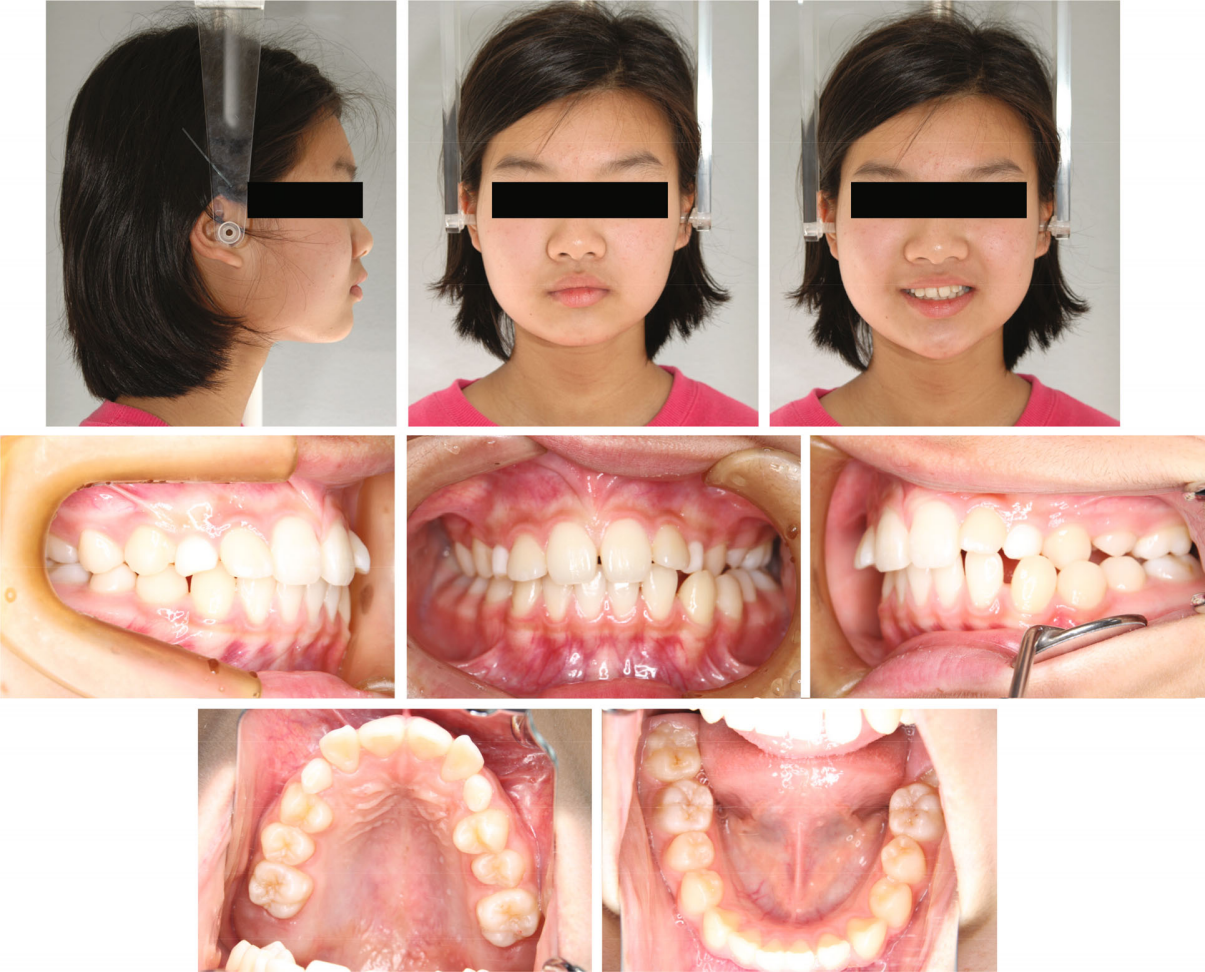
Pretreatment facial and intraoral photographs.

**Figure 2:**
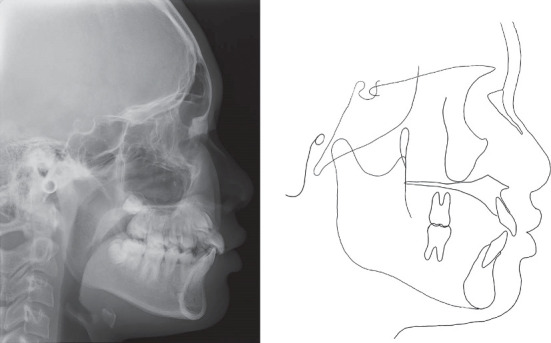
Pretreatment lateral cephalometric radiograph and cephalometric tracing.

**Figure 3:**
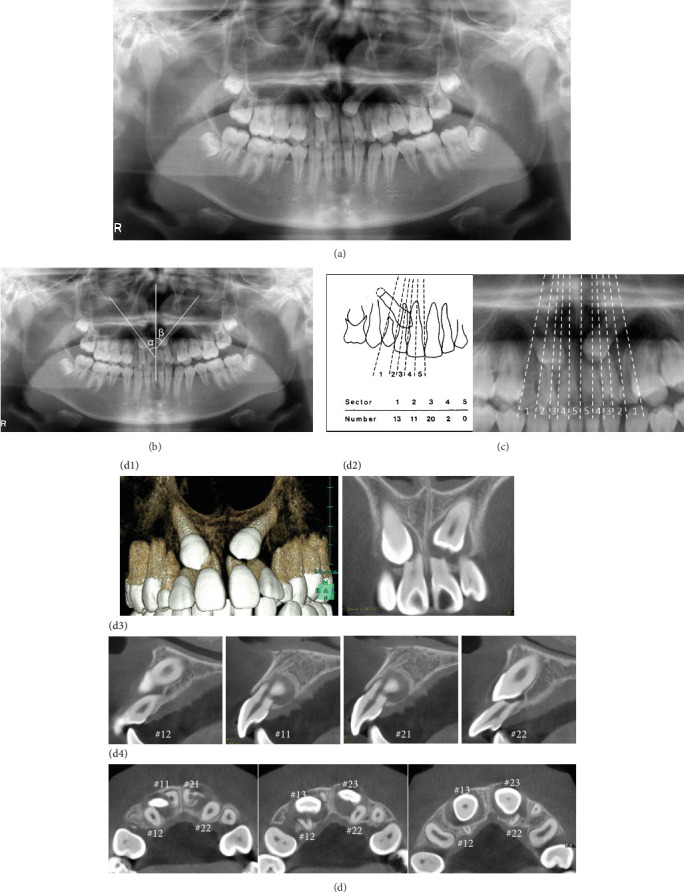
Pretreatment panoramic radiograph and cone-beam computed tomography (CBCT) images. (a) The impacted bilateral canines were mesially inclined. (b) The angle of inclination to the maxillary dental midline was 33° on the right side (*α*) and 43° on the left side (*β*). (c) According to Ericson and Kurol's classification, tooth 13 had grade 4 impaction and tooth 23 had grade 5 impaction. (d) Both canines were located on the buccal side and the roots of the four incisors were resorbed (D1; three dimensional CBCT image, D-2; coronal view, D-3; sagittal view, D-4; axial view).

An 11-year-old girl with impacted bilateral maxillary canines with an aberrant direction of eruption was referred to our department for orthodontic consultation. Root resorption of the four maxillary incisors had been detected on panoramic radiography at the previous dental clinic. She had no relevant medical and family history. The patient's general condition was good with regard to nutrition and psychosomatic development. Clinical examination revealed a bilateral Angle Class II molar relationship, overjet of 4.5 mm, overbite of 3.6 mm, crossbite on the left side, and bilateral deciduous canines. The contact between the permanent canines and roots of the lateral incisors caused labial inclination of the incisors (Figure 1). Cephalometric analysis revealed a skeletal Class I relationship (ANB angle 3.2°) and normal inclination of the angle of the upper and lower central incisors (U1 to SN angle 114.9°/FMIA 59.7°) (Figure 2 and Table 1). Panoramic radiography revealed that the bilateral maxillary canines were mesially inclined (Figure 3(a)), and the angle between the tooth axis and the maxillary dental midline was 33° on the right side and 43° on the left side (Figure 3(b)). According to Ericson and Kurol's classification 3, the medial displacement of the crown of the canine in relation to the long axis of the incisors reflected grade 4 impaction of tooth 13 (Federation Dentaire Internationale tooth numbering system) and grade 5 impaction of tooth 23 (Figure 3(c)). Cone-beam computed tomography showed that the canine crowns with cystic changes were located on the buccal aspect, between the maxillary bilateral central incisors, and that the lateral incisor roots were resorbed (Figure 3(d)).

Written informed consent was obtained from the patient and her parents. The statement of ethics approval by the ethics committee is not required as it is a single case report.

## 3. Diagnostic Summary

Diagnosis: Crowding with bilaterally impacted maxillary canines causing root resorption of the four maxillary incisors.

1. Angle Class II molar relationship.
2. Buccal cross bite on the left side of numbers 63, 24, 33, and 34.
3. Impacted maxillary bilateral permanent canines.
4. Deficient space for the maxillary canines in the maxillary arch.

## 4. Treatment Objectives

1. Surgical exposure and alignment of the bilateral maxillary canines.
2. Lateral crossbite correction.
3. Achievement of balanced and functional occlusion.

## 5. Course of Treatment

**Figure 4:**
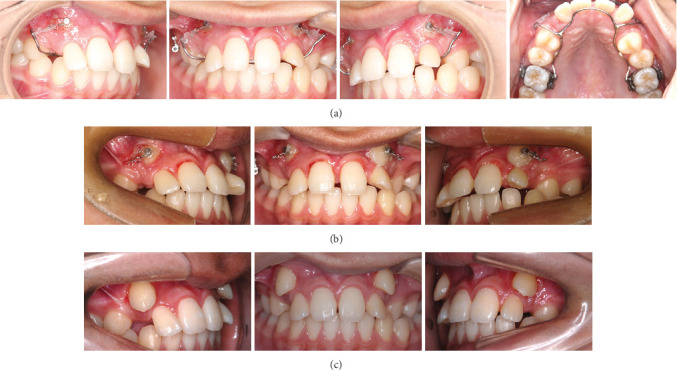
Treatment progress: (a) at the beginning of canine traction, (b) six months after canine traction, and (c) on completion of canine traction.

**Figure 5:**
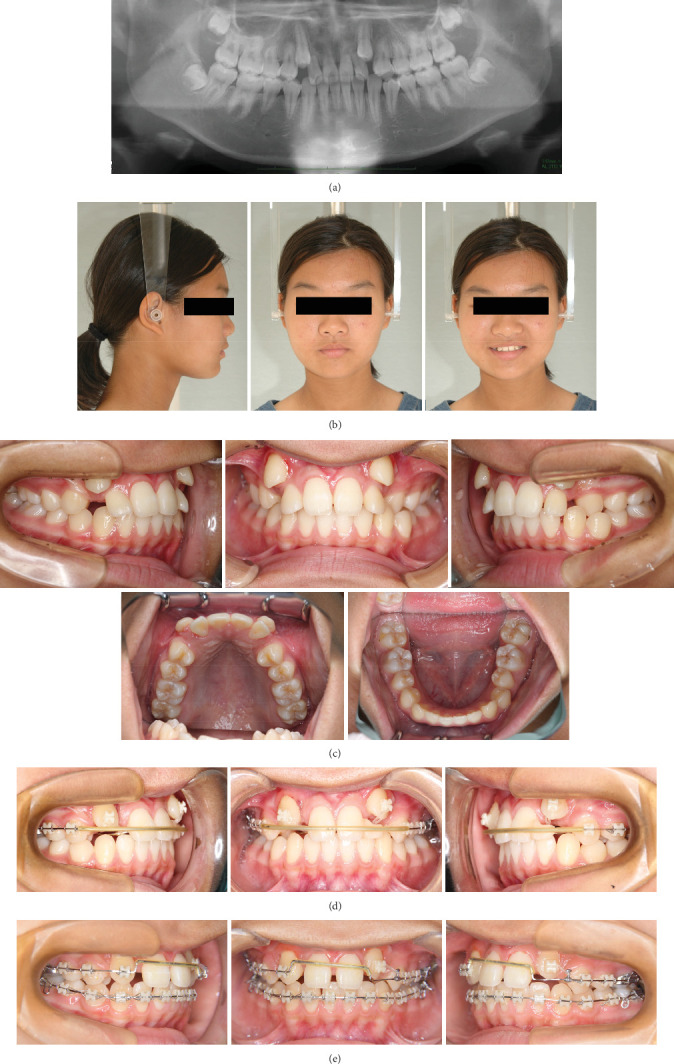
(a–c) Following completion of canine reposition. Facial and intraoral photographs and panoramic radiograph, (d and e) treatment progress.

**Figure 6:**
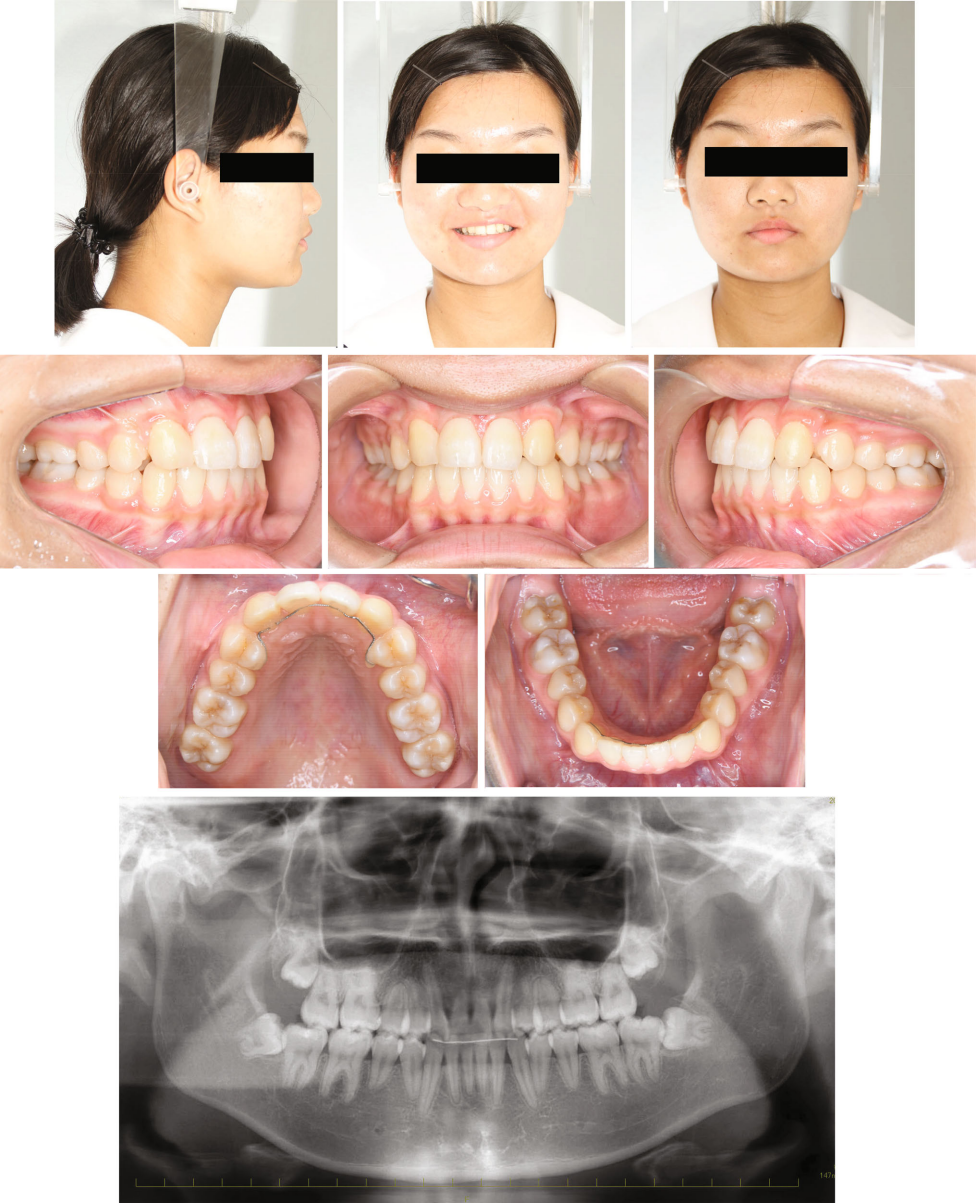
Posttreatment facial and intraoral photographs and panoramic radiograph.

**Figure 7:**
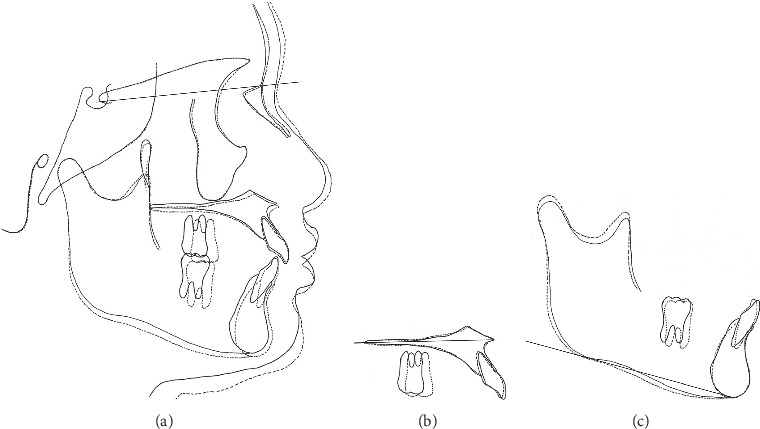
Superimposed tracing of the pretreatment and posttreatment lateral cephalometric radiographs: (a) overall, (b) maxilla, and (c) mandible.

**Figure 8:**
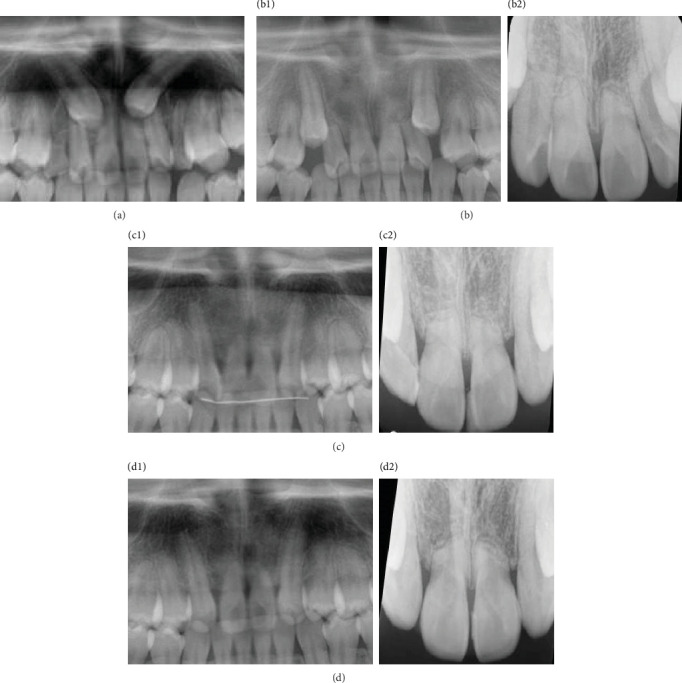
Panoramic radiograph revealing root resorption of maxillary incisors: (a) pretreatment, (b) post-canine traction, (c) posttreatment, and (d) two years posttreatment.

After extraction of the deciduous maxillary canines, both maxillary canines were surgically exposed. Full-thickness mucoperiosteal flaps were raised to gain access, and the soft tissue and bone around the crown were removed to facilitate orthodontic traction. The buttons attached to the ligature wire to tract the canines were bonded to the labial surface of the tooth. Initially the canines were tracted buccally to prevent further damage using elastics from the arm of the lingual arch. After confirming that part of the crown was exposed, the canines were tracted distally (Figures 4(a) and 4(b)). It took eight months to tract the maxillary canines to the correct vertical position, and the labial inclination of the lateral incisors improved spontaneously (Figure 4(c)). All incisors had Miller classification grade 2 mobility after the completion of canine traction. Panoramic radiography showed that the roots of the central and lateral incisors were equally resorbed (Figure 5(a)). After observing the mandibular growth for 6 months, multi-bracket treatment was initiated (Figures 5(b) and 5(c)). Extracting the lateral incisors was necessary to gain space for maxillary canine alignment. In addition, excessive force on the incisors must be avoided to prevent further root resorption. The canines, which were positioned superiorly, were pulled toward the occlusal plane with the hook of the lingual arch and arch-wire with elastic chains (Figure 5(d) and 5(e)). The crossbite on the left side was corrected, and all teeth were aligned with 0.018 × 0.25^″^ edgewise brackets. The treatment duration was 30 months. After achieving a well-balanced and functional occlusion (Figure 6), a wrap-around retainer was placed on the upper dentition, and the Hawley retainer was placed on the lower dentition. Superimposing the pre- and post-treatment cephalograms revealed that the treatment goals were mostly accomplished (Figure 7 and Table 1). Root resorption of the maxillary central incisors has not progressed for five years since the initial condition (Figure 8).

## 6. Discussion

For this report, a case of bilateral maxillary incisor root resorption caused by ectopic maxillary canines was presented. The maxillary canines deviate from their natural path of eruption more often than other teeth [11]. The maxillary canine tooth germ is located in a relatively crowded site in the maxilla, and the period between the generation of the tooth germ and completion of eruption is prolonged. Some studies have indicated that impacted canines and their ectopic formation might cause dental crowding, bony obstructions, tumors or cysts, and delayed replacement of deciduous canines [12, 13]. In this case, the patient had no relevant previous history, systemic factors, or local factors, such as trauma, inflammation, bad habits, or lesions in the maxilla. It is unclear why the canines had deviated from their natural path of eruption. It was speculated that the mesial malpositioning of the canines was a result of a lack of guidance.

In most cases, malpositioned canines avoid the incisor roots during eruption. Root resorption has been reported in approximately 12% of incisors adjacent to ectopic maxillary canines. Maxillary root resorption of permanent incisors was reported to occur in 48% of 9- to 15-year-old children with ectopic maxillary canine eruption [14]. The average angle of inclination of the maxillary canines on the panoramic radiograph was 18.6° in the group with root resorption and 29.5° in the group without [15]. There are few reports according to which central incisors, and not only the bilateral lateral incisors, can be affected. As stated in a previous report, in three out of 40 assessed patients, bilateral lateral incisors were affected by root resorption, however, none of the evaluated patients reported root resorption of all four incisors [16].

It was important to confirm the location and direction of erupted canines by radiography at the age of 10 years because no cases of root resorption have been observed before that age. However, in some cases, root resorption of the lateral incisors can extend to the pulp after the age of 10 years [17]. Early intervention to prevent malpositioning of maxillary canines could prevent severe root resorption of the maxillary incisor roots [18].

It was also reported that the extraction of deciduous canines in patients with displaced canines was an effective interceptive approach to correct ectopically erupting maxillary canines and prevent incisor root resorption [15]. In this case, if ectopic maxillary canines had been confirmed by panoramic radiography earlier and the deciduous canines had been extracted at the age of 10 years, the maxillary incisor root resorption may have been prevented.

Some studies have reported that when an ectopic maxillary canine is severely malpositioned in the horizontal and mesial direction, their extraction is considered because orthodontic traction may be a significant risk factor for incisor root resorption [18]. In this case, because the horizontal angle of the canines was not severe, traction and alignment of the bilateral canines after surgical exposure and extraction of the bilateral incisors with root resorption.

Many case reports indicate that orthodontically-induced resorption ceases when the force is removed; similarly, when an ectopic canine is maintained at an adequate distance from incisor roots, root resorption ceases [17, 19]. Furthermore, an apparent increase in maxillary incisor root resorption in cases of ectopic canines was not observed 10 years after orthodontic treatment [20]. For this case, the patient was advised to monitor the resorbed incisor as a precautionary measure. However, after consulting with a specialist, root canal treatment was deemed to be inappropriate as a means of reducing further resorption [21]. Therefore, it is necessary to evaluate the long-term status of teeth that have undergone root resorption during active orthodontic treatment.

## 7. Conclusions

This report described the case of an 11-year-old Japanese girl with root resorption of the bilateral central and lateral incisors, which was caused by impacted bilateral maxillary canines and maxillary crowding. Orthodontic treatment helped achieve the functional and esthetic goals through extraction of the bilateral lateral incisors with root resorption. Early detection and diagnosis through radiographic examinations could efficiently help prevent root resorption.

## Figures and Tables

**Table 1:** Superimposition of pre- and post-cephalograms.

Location	Measurement	Pretreatment	Posttreatment	Norm
Skeletal				
Maxilla to cranial base	SNA (°)	88.8	88.8	79.9 ± 2.4
Mandible to cranial base	SNB (°)	85.6	85.8	77.7 ± 2.7
Maxillomandibular	ANB (°)	3.2	3.0	2.9 ± 1.8
	FMA (°)	18.3	18.5	28.8 ± 5.6
Dental				
Maxillary dentition	U1 to SN (°)	114.9	118.7	104.5 ± 7.0
Mandibular dentition	IMPA (°)	102.1	93.4	91.3 ± 5.4
	FMIA (°)	59.7	68.1	59.9 ± 10.6
Intermaxillary dental relationship	Interincisal angle (°)	123.8	127.3	124.5 ± 11.6
